# Osteopontin and Osteoprotegerin as Potential Biomarkers in Abdominal Aortic Aneurysm before and after Treatment

**DOI:** 10.1155/2014/461239

**Published:** 2014-07-09

**Authors:** Konstantinos Filis, Vasilios Martinakis, George Galyfos, Fragiska Sigala, Dimitris Theodorou, Ioanna Andreadou, Georgios Zografos

**Affiliations:** ^1^Department of Propedeutic Surgery, Hippokration Hospital, University of Athens Medical School, Vasilissis Sofias 114 Avenue, 11527 Athens, Greece; ^2^Department of Pharmaceutical Chemistry, University of Athens School of Pharmacy, Vasilissis Sofias 114 Avenue, 11527 Athens, Greece

## Abstract

*Aim.* Although osteopontin (OPN) and osteoprotegerin (OPG) have been associated with abdominal aortic aneurysms (AAAs), no association of these two biomarkers with AAA surgical or endovascular treatment has been reported. *Material and Methods.* Seventy-four AAA patients were prospectively selected for open or endovascular repair. All aneurysms were classified (Types A–E) according to aneurysmal extent in CT imaging (EUROSTAR criteria). All patients had preoperative serum OPN and OPG values measurements and 1 week after the procedure. Preoperative and postoperative values were compared with a control group of twenty patients (inguinal hernia repair). *Results.* Preoperative OPN values in patients with any type of aneurysm were higher than in the control group, while OPG values showed no difference. Postoperative OPN values in AAA patients were higher than in the control group. OPN values increased after open surgery and after EVAR. OPG values increased after open surgery but not after EVAR. There was no difference in OPN/OPG values between EVAR and open surgery postoperatively. *Conclusions.* OPN values are associated with aneurysm presence but not with aneurysm extent. OPG values are not associated either with aneurysm presence or with aneurysm extent. OPN values increase after AAA repair, independently of the type of repair.

## 1. Introduction

Abdominal aortic aneurysms (AAAs) represent a chronic degenerative disease although the exact pathophysiological mechanism of AAA development seems to be obscure [1]. AAAs are characterized by chronic transmural inflammation, and they are associated with accumulation of monocytes/macrophages within the adventitia and media of the aortic wall [2–4]. Proinflammatory mediators (molecules) have an imperative role in stimulation and controlling of this inflammatory transmural infiltration [5].

OPN is a calcification inhibitor, expressed by many cell-types (osteoblasts, macrophages, and others) in response to biological stressors, and the regulation of its expression seems to play a key role in macrophages and vascular smooth muscle cells migration, linked to vascular remodeling and the development of atherosclerosis [6–8]. OPN plasma levels have been associated with the presence and the extentof cardiovascular disease independently of traditional risk factors [8–10]. On the other hand, OPG is a cytokine of the tumor necrosis factor receptor super-family and inhibits osteoclastogenesis [8, 11]. In the arterial system, smooth muscle cells and endothelial cells produce OPG, but its precise role in vascular pathophysiology remains undefined. Moreover, OPG has been positively associated with the presence and severity of coronary artery disease and the increased risk for cardiovascular diseases in general population [8, 12].

However, there has not been a study before comparing preoperative and postoperative OPN and OPG values in serum before and after AAA repair. The hypothesis tested in the present study was that open AAA repair (OR) and/or endovascular AAA repair (EVAR) possibly affect in a different way OPN or OPG serum levels, since (a) increased OPN and OPG values have been associated with the development and growth of AAAs [13–15] and (b) OR and EVAR affect differently the aneurysmal tissue exclusion from systemic arterial circulation. Studies so far have tried to correlate OPN and OPG values with the size of AAAs and specifically with the diameter of abdominal aortic aneurysms [13, 15]. In the present study, we further tried to examine the association of OPN and OPG serum levels with the anatomical extent of AAAs before and after treatment. Finally, our goal is to monitor the kinetics of both of these molecules after open and endovascular AAA treatment in order to produce conclusions regarding a potential utilization as biomarkers.

## 2. Material and Methods

From February 2008 to June 2012, out of 130 patients who were prospectively planned for elective abdominal aortic aneurysm repair (asymptomatic aneurysm with maximum diameter >50 mm), 74 AAA patients were screened for the study. Forty-five patients were excluded (see exclusion criteria) and 11 refused to participate. All AAA patients, except a full preoperative surgical assessment, had preoperative OPN and OPG serum values, as well as OPN and OPG values, 1 week postoperatively. Fasting blood samples were collected, centrifuged at 2500 rpm at 4°C, and stored at −80°C. OPN and OPG levels were determined by commercially available ELISA kits (RayBio Human OPN ELISA Kit Protocol (Cat. #: ELH-OPN-001) and RayBio Human OPG ELISA Kit Protocol (Cat. #: ELH-OPG-001) RayBiotech, Inc.). The sensitivity of the ELISA kit was minimally 50 pg/mL for OPN and 1 pg/mL for OPG detection, and the intra-assay and interassay coefficient of variation were <10%.

Exclusion criteria included active cancer, osteoporosis, recent transplantation, Crohn's disease, autoimmune diseases, severe coronary, carotid artery, or peripheral arterial disease [8, 16–20]. Evaluation of atherosclerotic arterial disease was performed by cardiac stress echo and—when indicated—coronary angiography (for coronary disease) and carotid-lower limb vascular duplex ultrasound (for peripheral vascular disease). The study was approved by the ethics committee of the hospital and informed consent from all patients was obtained.

All preoperative and postoperative OPN and OPG values were compared with OPN and OPG values of a control group that included twenty patients planned for a general surgery operation (inguinal hernia repair). All patients in the control group underwent abdominal ultrasonographic assessment in order to exclude the presence of an AAA. All patients in the control group had preoperative OPN and OPG serum values and OPN and OPG measurements 1 week postoperatively, as well.

Preoperative imaging of the AAAs included a CT angiography of the abdominal aorta and the common iliac arteries for all patients. According to the EUROSTAR trial criteria, all aneurysms were classified into 5 types (Types A–E), based on the anatomical extent of the aneurysm [21]. According to the above classification, all patients were further classified into two main groups: aortic aneurysms (Types A-B; extent from renal arteries to aortic bifurcation) and aortoiliac aneurysms (Types C–E). Out of the 74 patients, 34 patients underwent an OR and 40 patients underwent an EVAR. All open ORs were done using a specific standardized technique that included general anesthesia, transperitoneal approach by midline abdominal incision, resection of the anterior aneurysmal wall and contained thrombus, and insertion of the graft. Respectively, all EVARs were done using a specific standardized technique that included general anesthesia, catheterization of both femoral arteries after a cut-down incision in each groin, insertion of guiding wires, and introduction and deployment of the endograft. Comparisons of OPN and OPG levels between aortic aneurysms group versus aortoiliac aneurysms group and OR group versus EVAR group were performed as well.

Statistical analysis was carried out, using the *χ* ^2^ or Fisher exact tests, as appropriate, to compare proportions between the two different AAA repair methods and the two different groups of aneurysmal extent.* T*-tests were used for comparison of OPN and OPG values between the above groups. Results of statistical significance are reported with *P* values. *P* < 0.05 was considered significant for all analyses. Multivariate analysis was not pursued for any outcomes, because of low event rate.

## 3. Results

Out of the 74 patients with AAA included in the study, 78% had arterial hypertension and 81% dyslipidemia, while other risk factors, such as diabetes mellitus, chronic obstructive pulmonary disease, and smoking, showed a lower prevalence (Table 1). AAA patients and patients in the control group showed no differences regarding their demographic data, except from smoking (Table 1). Among the AAA group, 54 patients had an aortic aneurysm (Types A-B, EUROSTAR criteria) and 20 patients had an aortoiliac aneurysm (Types C–E, EUROSTAR criteria). Among the 54 patients with an aortic aneurysm, 81% had arterial hypertension and 78% dyslipidemia, while the other risk factors showed a lower prevalence. Among the 20 patients with an aortoiliac aneurysm, 70% had arterial hypertension and 90% dyslipidemia, while the other risk factors showed a lower prevalence as well. Demographic data between both groups of aneurysm extent did not show any statistical difference. OR group and EVAR group were similar concerning their demographic data as well.

### 3.1. Regarding Preoperative Levels of OPN and OPG

No difference was observed in preoperative OPG levels between the AAA group and the control group (Table 2). However, AAA patients showed higher preoperative OPN levels in comparison to control group (*P* = 0.017). OR group showed no different preoperative OPN and OPG levels compared to EVAR group. There was no difference between patients with an aortic aneurysm and patients with an aortoiliac aneurysm, concerning OPN and OPG levels. Both groups (aortic and aortoiliac) showed higher OPN levels, in comparison to the control group (aortic: *P* = 0.03 and aortoiliac: *P* = 0.01).

### 3.2. Regarding Postoperative Levels of OPN and OPG

There was no difference between postoperative and preoperative OPN and OPG levels in the control group. Both OPN (*P* = 0.001) and OPG (*P* = 0.01) increased in all AAA patients, in comparison to preoperative values. OPN levels in all patients increased after open surgery (*P* = 0.025) and after EVAR (*P* = 0.017) as well. OPN increased (*P* = 0.03) in patients with aortic aneurysms and in patients with aortoiliac aneurysms (*P* = 0.01), compared to preoperative values. OPN levels were further evaluated in subgroups (aortic or aortoiliac aneurysms in relation to OR or EVAR treatment) (Table 3).

Finally, postoperative OPN levels were compared between the different groups, as shown in Table 4. Postoperative OPN values in all AAA patients were higher than in the control group (*P* = 0.005). There was no difference in postoperative OPN values between patients treated by EVAR and patients treated by OR. Patients treated by OR showed higher postoperative OPN levels in serum, compared to the control group (*P* = 0.017). Patients treated by EVAR showed also higher postoperative OPN levels, compared to the control group (*P* = 0.0005). Patients with aortoiliac aneurysms showed higher postoperative OPN levels (*P* = 0.03), in comparison to patients with aortic aneurysm.

## 4. Discussion

This study has shown that plasma OPN levels are increased in patients with AAA compared to a matched group of patients without AAA. However, OPN levels are not associated with aneurysm extent. Furthermore, OPN is increased after AAA repair either by open surgery or endovascular grafting, although it is not increased after inguinal hernia repair. Type of AAA repair (OR or EVAR) does not affect postoperative OPN increase differently. Finally, OPG levels are not associated with the presence of AAA, AAA extent, and type of AAA treatment.

Osteopontin has been associated with changes in the extracellular matrix and depletion of vascular smooth muscle cells, leading to AAA development [2, 4, 22]. Our results concur with previous studies showing that OPN is associated with AAA formation and aortic dilatation as well [13, 23]. Furthermore, there were previous researchers, such as Golledge et al., that correlated OPN serum levels with AAA growth and size [13, 24]. However, in our study we focused only on the anatomic extent of large (>50 mm in diameter) aneurysms. We have shown that aortic and aortoiliac abdominal aneurysms do not show any difference in their serum OPN levels. Based on our data and Golledge's data, we may hypothesize that OPN is not produced by the aneurysmal aortic tissue itself, but it may play an essential pathogenetic role in aneurysm development.

Many studies have correlated OPN and OPG levels with arterial atheromatosis/calcification and presence of aneurysm as well [8, 25–27]. For the first time, however, we tried to examine possible changes of OPN and OPG serum levels after open AAA repair or EVAR, in comparison to preoperative values. In a recent study, OPN levels were measured in serum before and after coronary artery by-pass grafting (CABG), and a decrease of OPN levels was observed postoperatively [28]. The relationship between plasma OPN levels and left-ventricular volume and function in 18 consecutive patients who underwent successful reperfusion after acute myocardial infarction was evaluated in another study [29]. The plasma osteopontin level was within the control range at admission, began to increase on day 2, and reached a maximum around day 3. These data have shown that OPN is rapidly increased after myocardial infarction and then decrease to the initial levels after 6-7 days. In our study, we have chosen to measure OPN 7 days posttreatment, so as to ameliorate the possible effect of surgical stress on OPN and OPG levels. These levels were unaffected by surgery on day 7 in the control group. Furthermore, early inflammatory response after any type of AAA treatment is minimized one week after the repair [30].

One hypothesis tested in our study was that, since a part of the aortic wall and the contained thrombus is incised and removed (by OR), serum OPN levels had to decrease, provided that aneurysmal wall and/or thrombus react either as producers or as promoters of OPN. The results showed, on the contrary, that OPN levels increase after AAA repair in all patients, treated either by OR or by EVAR. It is of great interest that postoperative OPN levels after EVAR were higher in patients with aortoiliac aneurysm, in comparison to patients with aortic aneurysm, while OPN after open AAA repair did not show such a difference. The significance of these data is almost impossible to be explained with the available scientific evidence from other studies and the underlying relative mechanisms are not completely understood.

However, our study presents new data that may enhance future studies to compare the clinical role of OPN as a biomarker for follow-up after different type of treatments (in open AAA repair most of the aneurysm and thrombus are removed, while by EVAR all aneurysmal tissue and thrombus remain around the endograft). Possibly, future research has to focus on measuring the aortic tissue OPN production and/or the aneurysmal thrombus OPN production, in order to differentiate if OPN is an aneurysm end-product or an initial key player in aneurysm pathogenesis. Moreover, new studies could investigate a potential utilization of OPN as a prognostic marker for occurrence of para-anastomotic aneurysms after open repair or neck dilatation after EVAR.

Regarding osteoprotegerin, studies have shown that it stimulates autophagy via important signaling pathways in vascular smooth muscle cells, leading this way to the weakening and remodeling of the arterial wall [31]. Therefore, more than one study conclude that OPG serum values are associated with the presence and progression of AAAs [15, 32]. Moreover, Koole et al. proved that OPG is associated with aneurysm diameter and proteolysis in abdominal aortic aneurysm disease [33]. Our study, however, showed that serum OPG levels are not associated either with presence of AAA or with the aneurysm extent.

Limitations in our study included the relative small number of patients, who underwent open AAA repair or EVAR, and the small number of patients in the control group, as well. This disadvantage did not allow for further subgroup analyses, according to gender, age, or other patient characteristics. Moreover, a protocol of serial OPN and OPG measurements on the immediate postoperative days would be more appropriate, in order to conclude the exact variation of OPN levels after AAA treatment. In addition, although we excluded from the study all patients with severe coronary, carotid, or peripheral arterial disease, OPN has been detected in all stages of atherosclerosis and, therefore, comparisons among groups may carry an error related to nonaneurysmal atherosclerotic disease.

The clinical significance of OPN/OPG levels determination in AAA is not obvious yet. It should be emphasized that there is at least one in vitro study so far to suggest that OPN is downregulated by peroxisome proliferator-activated receptor (PPAR) ligation [24]. Those findings in association with our results justify the need for new studies to reevaluate the role of OPN in a possible nonoperative therapy of abdominal aortic aneurysms in the future.

## 5. Conclusions

In conclusion, circulating levels of plasma OPN may indicate an independent prognostic factor for AAA formation, while the role of OPG levels remains questionable. Open surgery and endovascular grafting are followed by an increase of OPN after one week. We need larger studies to establish if there is a possible prognostic value of OPN during the period of aneurysm follow-up, before and after surgical or endovascular treatment, and its clinical significance.

## Figures and Tables

**Table 1:** Demographic data of control Group and AAA patients (overall, OR, and EVAR groups). All values of statistical significance in parenthesis are versus the control group.

	AAA patients			Control group (*n* = 20)
	Overall (*n* = 74)	OR (*n* = 34)	EVAR (*n* = 40)	
Arter. hypertension (%)	58 (78%) (NS)	29 (78%) (NS)	29 (73%) (NS)	17 (85%)
Dyslipidemia (%)	60 (81%) (NS)	28 (82%) (NS)	32 (80%) (NS)	13 (65%)
D. mellitus (%)	10 (14%) (NS)	4 (12%) (NS)	6 (15%) (NS)	3 (15%)
COPD (%)	8 (11%) (NS)	4 (12%) (NS)	4 (10%) (NS)	2 (10%)
Smoking (%)	50 (68%) (*P* = 0.037)	24 (71%) (*P* = 0.044)	26 (65%) (NS)	8 (40%)
Middle age (years) ± SD	65.3 ± 8 (NS)	64.2 ± 6 (NS)	66.5 ± 5 (NS)	64.7 ± 8
Male gender (%)	52 (68%) (NS)	23 (71%) (NS)	29 (73%) (NS)	13 (65%)

AAA: aortic abdominal aneurysm; OR: open repair; EVAR: endovascular aneurysm repair; COPD: chronic obstructive pulmonary disease.

**Table 2:** Serum OPN and OPG correlations of AAA patients (overall, aortic, and aortoiliac aneurysm groups) versus the control group and OR versus EVAR group. All mean values are given with standard deviation (SD).

	AAA patients			Control group (*n* = 20)
	Overall (*n* = 74)	Aortic (*n* = 54)	Aortoiliac (*n* = 20)	
OPN (mean value ± SD, ng/mL)	3661.64 ± 1126.49 (*P* = 0.017)	3878.30 ± 1381.69 (*P* = 0.03)	3076.68 ± 1778.5 (*P* = 0.01)	1418.30 ± 962.68
OPG (mean value ± SD, ng/mL)	354.28 ± 212.1 (NS)	330.69 ± 187.45 (NS)	417.97 ± 262.64 (NS)	364.87 ± 159.85

	OR (*n* = 34)		EVAR (*n* = 40)	*P*

OPN (mean value ± SD, ng/mL)	2194.15 ± 800.24		4909.01 ± 2574.6	NS
OPG (mean value ± SD, ng/mL)	320.96 ± 206.6		382.6 ± 215.17	NS

AAA: aortic abdominal aneurysm; OR: open repair; EVAR: endovascular aneurysm repair; OPN: osteopontin; OPG: osteoprotegerin.

**Table 3:** Comparison of preoperative and postoperative mean values of osteopontin (OPN) and osteoprotegerin (OPG) in different groups of patients. All values are presented with standard deviation (SD) and units are in ng/mL.

	Preoperative values (ng/mL)	Postoperative values (ng/mL)	*P*	Groups
OPN	3661.64 ± 1126.49	14893.13 ± 3446.5	0.001	AAA patients
OPG	354.28 ± 212.1	409.28 ± 145.6	0.01	

OPN	2194.15 ± 800.24	15170.79 ± 3798.63	0.025	OR
OPG	320.96 ± 206.6	397.74 ± 204.7	0.02	

OPN	4909.01 ± 2574.6	14657.13 ± 2805.65	0.017	EVAR
OPG	382.60 ± 215.17	419.09 ± 195.78	NS	

OPN	2086.40 ± 769.8	15767.60 ± 3689.67	0.04	Aortic (open repair)
OPG	270.60 ± 167.5	321.52 ± 156.43	0.02	

OPN	2697 ± 1056.8	12585.80 ± 2897.34	0.045	Aortoiliac (open repair)
OPG	556.20 ± 269.89	753.41 ± 387.5	0.03	

OPN	5808 ± 3045.6	6938.12 ± 3867.5	NS	Aortic (EVAR)
OPG	395.40 ± 205.43	417.23 ± 167.34	NS	

OPN	3239.40 ± 989.6	28992.03 ± 7809.67	0.02	Aortoiliac (EVAR)
OPG	358.70 ± 187.78	429.90 ± 148.9	NS	

OPN	3878.30 ± 1381.69	11516.50 ± 2498.56	0.03	Aortic (both types of repair)
OPG	330.69 ± 187.45	367.51 ± 188.4	0.02	

OPN	3076.68 ± 1778.5	24010 ± 6794.75	0.01	Aortoiliac (both types of repair)
OPG	417.97 ± 262.64	518.06 ± 289.54	NS	

AAA: aortic abdominal aneurysm; OR: open repair; EVAR: endovascular aneurysm repair; OPN: osteopontin; OPG: osteoprotegerin.

**Table 4:** Comparison of postoperative OPN and OPG values between different groups of patients.

	Postoperative values (ng/mL)		*P*
	Control group	AAA patients	
OPN	964.06 ± 468.5	14893.13 ± 3446.5	0.005
OPG	350.28 ± 112.89	409.28 ± 145.6	NS

	Open repair	EVAR	*P*

OPN	15170.79 ± 3798.63	14657.13 ± 2805.65	NS
OPG	397.74 ± 204.7	419.09 ± 195.78	NS

	Control group	Open repair	*P*

OPN	964.06 ± 468.5	15170.79 ± 3798.63	0.017
OPG	350.28 ± 112.89	397.74 ± 204.7	NS

	Control group	EVAR	*P*

OPN	964.06 ± 468.5	14657.13 ± 2805.65	0.0005
OPG	350.28 ± 112.89	419.09 ± 195.78	NS

	Aortic	Aortoiliac	*P*

OPN	11516.50 ± 2498.56	24010 ± 6794.75	0.03
OPG	367.51 ± 188.4	518.06 ± 289.54	NS

	Aortic (open repair)	Aortoiliac (open repair)	*P*

OPN	15767.60 ± 3689.67	12585.80 ± 2897.34	NS

	Aortic (EVAR)	Aortoiliac (EVAR)	*P*

OPN	6938.12 ± 3867.5	28992.03 ± 7809.67	0.003

AAA: aortic abdominal aneurysm; OR: open repair; EVAR: endovascular aneurysm repair; OPN: osteopontin; OPG: osteoprotegerin.
